# A new malaria vector mosquito in South Africa

**DOI:** 10.1038/srep43779

**Published:** 2017-03-06

**Authors:** Ashley Burke, Leonard Dandalo, Givemore Munhenga, Yael Dahan-Moss, Frans Mbokazi, Sifiso Ngxongo, Maureen Coetzee, Lizette Koekemoer, Basil Brooke

**Affiliations:** 1 Wits Research Institute for Malaria, School of Pathology, Faculty of Health Sciences, University of the Witwatersrand, Johannesburg, South Africa; 2 Centre for Opportunistic, Tropical & Hospital Infections, National Institute for Communicable Diseases, Johannesburg, South Africa; 3 Malaria Elimination Programme, Mpumalanga Department of Health, Ehlanzeni District, South Africa; 4 Environmental Health, Malaria and Communicable Disease Control, KwaZulu-Natal Department of Health, South Africa

## Abstract

South Africa aims to eliminate malaria within its borders by 2018. Despite well-coordinated provincial vector control programmes that are based on indoor residual insecticide spraying, low-level residual malaria transmission continues in the low-altitude border regions of the north-eastern sector of the country. In order to identify the underlying causes of residual transmission, an enhanced vector surveillance system has been implemented at selected sites in the Mpumalanga and KwaZulu-Natal (KZN) provinces. The collection periods for the data presented are March 2015 to April 2016 for Mpumalanga and January 2014 to December 2015 for KZN. The mosquito collection methods used included indoor and outdoor traps based on the use of traditional ceramic pots, modified plastic buckets and exit window traps (KZN only). All *Anopheles funestus* species group mosquitoes collected were identified to species and all females were screened for the presence of *Plasmodium falciparum* sporozoites. Two *An. vaneedeni* females, one from each surveillance site, tested positive for *P. falciparum* sporozoites. These are the first records of natural populations of *An. vaneedeni* being infective with *P. falciparum*. As both specimens were collected from outdoor-placed ceramic pots, these data show that *An. vaneedeni* likely contributes to residual malaria transmission in South Africa.

Malaria is a parasitic disease caused by *Plasmodium* protozoa and transmitted by female *Anopheles* mosquitoes (Diptera: Culicidae)^1^. The populations most at risk live in sub-Saharan Africa which accounts for 80% of cases and 90% of total deaths^2^.

Malaria transmission in South Africa is limited to the low-altitude northern and north-eastern border regions of the country which span the Limpopo, Mpumalanga and KwaZulu-Natal (KZN) provinces^3^. Historically, only the major malaria vector *Anopheles funestus* was directly implicated in malaria transmission in South Africa^4^. In addition, the malaria vectors *An. arabiensis* and *An. merus* were provisionally implicated based on their occurrence in South Africa’s malaria affected regions and because they have been directly implicated in transmission in neighbouring southern Mozambique^5^,^6^,^7^,^8^. Recently, several *An. arabiensis* and one *An. merus* specimen collected outdoors during 2014–2016 were also found to be infected with *P. falciparum* sporozoites in KwaZulu-Natal (Dandalo *et al*., submitted), thus expanding the number of species directly implicated in malaria transmission within South Africa.

Malaria vector control in South Africa’s malaria affected provinces is primarily based on indoor spraying of long-lasting residual insecticides^8^. The indoor residual spraying (IRS) method has been the mainstay of malaria vector control in South Africa since the 1940s and has remained effective owing to carefully co-ordinated provincial IRS programmes^9^. Despite this, low-level residual malaria transmission continues and is likely caused by outdoor feeding and resting *Anopheles* vector mosquitoes that are unaffected by indoor applications of insecticide^10^. Maintaining effective control whilst scaling up control methods to address ongoing residual malaria transmission within South Africa are high priority activities because the country has adopted a malaria elimination agenda and aims to eliminate malaria within its borders by 2018^11^.

Continuing residual malaria transmission and the burgeoning incidence of insecticide resistance in malaria vector populations within South Africa’s borders^4^,^12^ have necessitated an intensification of vector surveillance activities in the affected provinces. The principle objectives of these enhanced surveillance activities are to compare and establish optimal methods of collecting adult *Anopheles* mosquitoes, to establish which *Anopheles* species are responsible for ongoing residual malaria transmission, to assess the extent of residual malaria transmission within South Africa and to assess the range and geographical extent of insecticide resistance in incriminated vector populations. Within these broad objectives, the aim of this project was to assess whether *An. vaneedeni*, a member of the *An. funestus* species group^13^, contributes to residual malaria transmission in South Africa.

## Results

**Figure 1:**
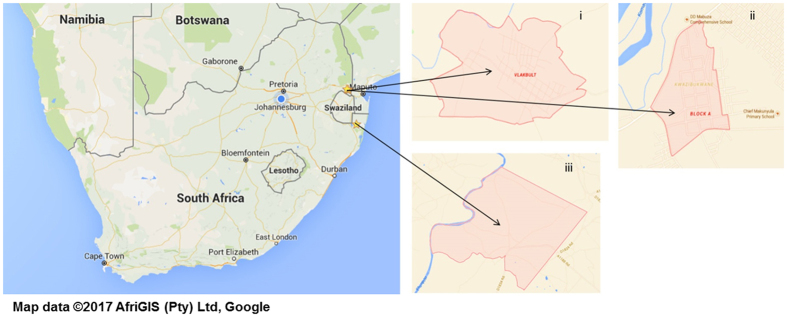
*Anopheles* mosquito surveillance sites at Vlakbult (i) and Block A (ii) (Ehlanzeni District of Mpumalanga) and Mamfene (iii) (KwaZulu-Natal) South Africa. Map source data were obtained from Map data (c) 2016 AfriGIS (Pty) Ltd, Google (https://www.google.co.za/maps/place/South+Africa/).

*Anopheles* vector surveillance for indoor and outdoor-resting mosquitoes was conducted in two villages in Mpumalanga Province (Tonga - Block A and Vlakbult) (S25°42′03″; E31°48′31″ and S25°38′42″; E31°42′01″) for one year (March 2015–April 2016) and in Mamfene, KwaZulu-Natal (KZN) Province (S27°23′50.5″; E032°12′20.1″) for two years (January 2014–December 2015) (Fig. 1).

A total of 255 and 77 *An. funestus* group specimens was collected from the KZN and Mpumalanga sites respectively (Table 1). Of these, 45 of the adult females collected from Block A and Vlakbult (Mpumalanga) and 51 of the adult females collected from Mamfene (KZN) were positively identified as *An. vaneedeni*, first by morphology^10^ to *An. funestus* group followed by PCR^14^ to species (Table 1). Two of these *An. vaneedeni* specimens, one from each province, tested positive for the presence of *Plasmodium falciparum* sporozoites through two ELISA assays. These data give *P. falciparum* infectivity rates for *An. vaneedeni* of 2.44% and 1.96% for the Mpumalanga and KZN sites respectively.

The *Anopheles* species identification of these two specimens as *An. vaneedeni* was further confirmed by sequencing their internal transcribed spacer 2 (ITS2) region^14^. There was a 99% sequence identity between the ITS2 region of the KZN and Mpumalanga specimens and the published *An. vaneedeni* sequence originating from South Africa (GenBank accession number JN994152.1^15^). The presence of *P. falciparum* sporozoite DNA was confirmed by nested PCR for the KZN specimen^16^. Sequence analysis of the nested PCR product revealed that there was a 99% identity to the published sequence for *P. falciparum* (GenBank accession number KT991235.1^17^).

None of the *An. leesoni, An. rivulorum* and *An. parensis* female specimens from either field site (Table 1) showed positive for *P. falciparum* sporozoites based on ELISA analysis.

## Discussion

The perennial occurrence of several *An. funestus* species group members–*An. vaneedeni, An. rivulorum, An. leesoni* and *An. parensis*–at the Mpumalanga and KwaZulu-Natal field sites warrants investigation into their possible contribution to malaria transmission in these regions, especially given that *An. rivulorum* has been implicated in malaria transmission in Tanzania^18^ and Kenya^19^, and a previous survey has shown that *An. parensis* will readily rest indoors in the Mamfene region^20^. The evident absence of *An. funestus sensu stricto* at these sites can be attributed to ongoing annual IRS based control activities, particularly the use of DDT which has effectively eradicated this species from South Africa. This is because *An. funestus* in South Africa is highly susceptible to DDT^4^,^8^ and has a strong tendency to rest indoors, making this species especially susceptible to IRS programmes that utilize DDT^7^,^9^.

**Figure 2:**
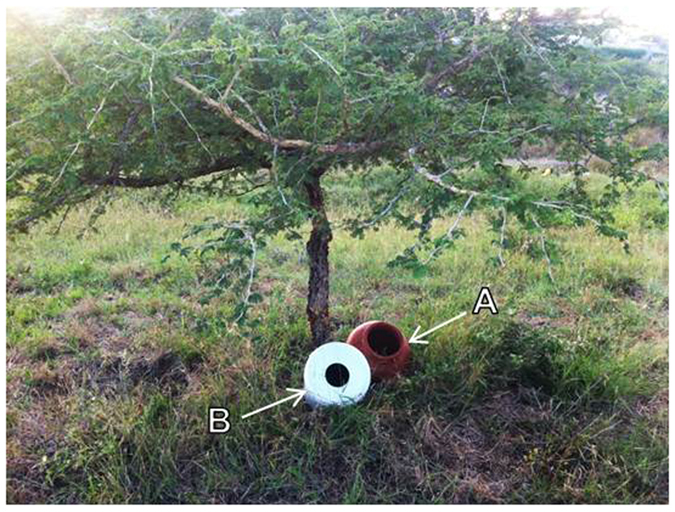
Ceramic pot (**A**) and modified plastic bucket (**B**) used for adult *Anopheles* mosquito surveillance, Mpumalanga and KwaZulu-Natal Provinces, South Africa.

The data summarised here represent the first record of wild-caught *P. falciparum* sporozoite positive *An. vaneedeni* females, directly implicating this species in malaria transmission in South Africa. Although this species is considered to be to be primarily zoophilic^10^, it will readily feed on humans outdoors and has previously been experimentally infected with *P. falciparum* under laboratory conditions^21^. The outdoor-resting and feeding traits of this species are reinforced by the fact that most of the *An. vaneedeni* specimens collected in these surveys, including the two that tested sporozoite positive, were found in outdoor-placed ceramic pots (Fig. 2) deployed at randomly selected households at the two sites.

The geographical range of *An. vaneedeni* primarily includes the north-eastern low-altitude regions of South Africa, and likely extends into southern and eastern Zimbabwe and southern Mozambique^22^. However, there are collection records of *An. vaneedeni* in the western highlands of Kenya^23^, suggesting that its range may be substantially more extensive.

The collection of sporozoite-positive, outdoor-resting *An. vaneedeni* supports the hypothesis of ongoing outdoor residual malaria transmission in South Africa, as first proposed by De Meillon *et al*. in ref. 21, and tentatively suggests that this species may also be contributing to malaria transmission in other malaria-endemic countries in which it occurs. This information highlights the need to intensify malaria vector control in South Africa by including methods designed to target outdoor feeding vector populations without compromising the efficacy of the IRS programme.

## Materials and Methods

### Ethical statement

Informed consent was obtained from all household owners involved in this study. Ethical clearance for the collection of mosquito specimens was obtained from the University of the Witwatersrand (M141023 & W-CJ-150520-2) and the KwaZulu-Natal Department of Health (HRKM337/14).

### Mosquito collections

**Figure 3:**
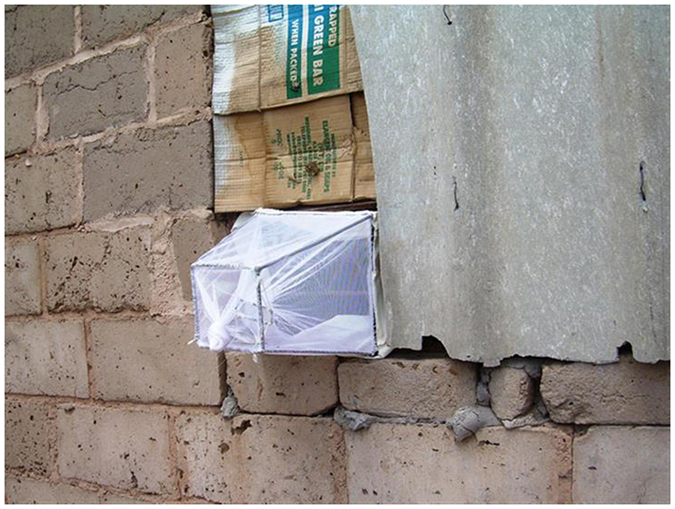
Window exit trap used for adult *Anopheles* mosquito surveillance, KwaZulu-Natal Province, South Africa.

Adult *Anopheles* mosquitoes were collected using traditional ceramic pots and modified plastic buckets (Fig. 2). These were placed both inside and outside selected households in Vlakbult and Block A in Mpumalanga (March 2015–April 2016), and only outside at households in Mamfene, KZN (January 2014–December 2015). Traps were not deployed indoors at Mamfene because homeowners consent did not include this provision and because exit window traps were instead used to collect indoor resting mosquitoes at this site (Fig. 3). The traps were cleared at sunrise weekly by the malaria vector surveillance teams based near the collection sites in each province.

### *Anopheles* species identification and vector incrimination

All *Anopheles* specimens collected were preserved on silica and initially identified by external morphology using dichotomous keys^10^. Those identified as belonging to the *An. funestus* group were subsequently identified to species level by PCR^14^. All females were screened for the presence of *Plasmodium falciparum* sporozoites by ELISA^24^. *Plasmodium falciparum* infectivity, where indicated by ELISA, was confirmed with a nested *Plasmodium* PCR^16^.

### Sequence analysis of mosquito ITS2 and *Plasmodium falciparum* ssRNA

In order to confirm *Anopheles* species identity, the internal transcribed spacer region 2 (ITS2) region of the rDNA of each *An. vaneedeni* sporozoite positive sample was amplified using the following primers: ITS2A: 5′-TGTGAACTGCAGGACA-CAT-3′; and ITS2B: 5′-TATGCTTAAATTCAGGGGGT-3′. PCR conditions were the same as those used in the *An. funestus* species-specific PCR^14^. In order to confirm *Plasmodium* species identity, *Plasmodium* ssRNA from the KwaZulu-Natal *An. vaneedeni* sporozoite positive sample was amplified using the following primers: rPLU5: 5′-CCTGTTGTTGCCTTAAACTTC-3′; rPLU6: 5′-TTAAAATTGTTGCAGTTAAAACG-3 for the first amplification step, and rFAL1: 5′-TTAAACTGGTTTGGGAAAACCAAATATATT-3′; and rFAL2: 5′-ACACAATGAACTCAATCATGACTACCCGTC-3′ for the second amplification step and sequencing. PCR conditions were the same as those previously described^16^. All amplicons were electrophoresed on 2.5% agarose gels stained with ethidium bromide and product sizes were confirmed using a molecular weight marker (Thermo Scientific O’GeneRuler 100 bp DNA Ladder; 0.1 μg/μl concentration, supplied with 1Ml 6x Orange DNA Loading Dye). ITS2 PCR products were purified and sequenced by Macrogen. The sequences were manually edited by BioEdit version 7.2.5^25^. Subsequently, the sequences were aligned with sequences stored in GenBank using nucleotide BLAST (BLASTn) (http://blast.ncbi.nlm.nih.gov/Blast.cgi).

## Additional Information

**How to cite this article:** Burke, A. *et al*. A new malaria vector mosquito in South Africa. *Sci. Rep.* **7**, 43779; doi: 10.1038/srep43779 (2017).

**Publisher's note:** Springer Nature remains neutral with regard to jurisdictional claims in published maps and institutional affiliations.

## Figures and Tables

**Table 1:** Distribution of *Anopheles funestus* group collected by species and gender from the Mpumalanga (Vlakbult and Block A: March 2015–April 2016) and KwaZulu-Natal (Mamfene: January 2014–December 2015) *Anopheles* mosquito surveillance sites, South Africa.

Province	Site	Species	Total	
			Males	Females
Mpumalanga	Vlakbult	*An. vaneedeni*	11	41
		*An. rivulorum*	1	3
		*An. leesoni*	0	2
	Block A	*An. vaneedeni*	0	3
		*An. rivulorum*	0	16
		*An. leesoni*	0	0
KwaZulu-Natal	Mamfene	*An. vaneedeni*	25	51
		*An. rivulorum*	5	13
		*An. leesoni*	13	53
		*An. parensis*	34	61
